# Inference of gene regulatory networks from genome-wide knockout fitness data

**DOI:** 10.1093/bioinformatics/bts634

**Published:** 2012-12-27

**Authors:** Liming Wang, Xiaodong Wang, Adam P. Arkin, Michael S. Samoilov

**Affiliations:** ^1^Department of Electrical and Computer Engineering, Duke University, Durham, NC 27708, USA, ^2^Department of Electrical Engineering, Columbia University, New York, NY 10027, USA, ^3^Department of Bioengineering, University of California Berkeley, Berkeley, CA 94720, USA and ^4^Physical Biosciences Division, Lawrence Berkeley National Laboratory, Berkeley, CA 94720, USA

## Abstract

**Motivation:** Genome-wide fitness is an emerging type of high-throughput biological data generated for individual organisms by creating libraries of knockouts, subjecting them to broad ranges of environmental conditions, and measuring the resulting clone-specific fitnesses. Since fitness is an organism-scale measure of gene regulatory network behaviour, it may offer certain advantages when insights into such phenotypical and functional features are of primary interest over individual gene expression. Previous works have shown that genome-wide fitness data can be used to uncover novel gene regulatory interactions, when compared with results of more conventional gene expression analysis. Yet, to date, few algorithms have been proposed for systematically using genome-wide mutant fitness data for gene regulatory network inference.

**Results:** In this article, we describe a model and propose an inference algorithm for using fitness data from knockout libraries to identify underlying gene regulatory networks. Unlike most prior methods, the presented approach captures not only structural, but also dynamical and non-linear nature of biomolecular systems involved. A state–space model with non-linear basis is used for dynamically describing gene regulatory networks. Network structure is then elucidated by estimating unknown model parameters. Unscented Kalman filter is used to cope with the non-linearities introduced in the model, which also enables the algorithm to run in on-line mode for practical use. Here, we demonstrate that the algorithm provides satisfying results for both synthetic data as well as empirical measurements of *GAL* network in yeast *Saccharomyces cerevisiae* and *TyrR–LiuR* network in bacteria *Shewanella oneidensis*.

**Availability:** MATLAB code and datasets are available to download at http://www.duke.edu/∼lw174/Fitness.zip and http://genomics.lbl.gov/supplemental/fitness-bioinf/

**Contact:** wangx@ee.columbia.edu or mssamoilov@lbl.gov

**Supplementary information:** Supplementary data are available at *Bioinformatics* online

## 1 INTRODUCTION

In recent years, modelling and inference of biological regulatory networks have become an active area of research in large part owing to the emergence of microarray technology, which allows for simultaneous measurement of gene expression on the genome-wide scale (Bonneau *et al.*, 2006; Chou and Voit, 2009; Friedman *et al.*, 2000; Liang and Wang, 2008; Margolin *et al.*, 2006; Reiss *et al.*, 2006; Shmulevich *et al.*, 2002; Stuart *et al.*, 2003). The vast amounts of data provided by gene expression microarrays enable the possibility of accurate estimation of gene regulatory network organization, which has greatly benefited a broad range of disciplines—from basic biological sciences, to bioengineering, to medical diagnosis and treatment (Hanai *et al.*, 2006; Mischel *et al.*, 2004). The goal of inference algorithms is to discover the connectivity structure and, potentially, dynamic characteristics of these networks based on such time- or other state-series data. Among other things, the nature of inference algorithms varies depending on the types of biological networks and the way they are modelled (de Jong, 2002; Hendrickx *et al.*, 2011; Lecca *et al.*, 2011; Liu *et al.*, 2006; Samoilov *et al.*, 2001; Shmulevich *et al.*, 2002; Tian and Burrage, 2003; Wang and Schonfeld, 2010). One category of models quantizes the empirical data into binary numbers and views network structures as Boolean constraints (Bornholdt, 2008; Kauffman *et al.*, 2003). Although this could be attempted in a deterministic framework, both the uncertainties introduced by measurement errors as well as the inherent stochasticity of gene expression make any experimental data substantially probabilistic. To impart this random nature to the Boolean framework, the probability Boolean network models have been introduced (Akutsu *et al.*, 1999; Huang, 1999; Shmulevich *et al.*, 2002). However, as biological processes are neither digital nor homogeneous, further gene regulatory modelling and inference refinements may be achieved by using alternative probabilistic network descriptions (Craciun *et al.*, 2013; Kellam *et al.*, 2002; Liu *et al.*, 2006), continuous-time differential equations (Chen *et al.*, 1999; Holter *et al.*, 2001; Wang *et al.*, 2008), stochastic differential equations (Tian and Burrage, 2003; Yeung *et al.*, 2002), and control theory methods (Beal *et al.*, 2005; Cook *et al.*, 1998; Rangel *et al.*, 2004), among others. Although any of these methods offers certain advantages and disadvantages in attempting to capture the structure and dynamics of gene regulatory network, it should be noted that they have largely been designed toward describing gene expression data.

Recently, however, a new type of high-throughput data has emerged and seen rapid proliferation in empirical biosciences—the *genome-wide fitness data*. At its core, this involves using latest technological advances to massively scale the traditional gene deletion/interruption studies in order to achieve nearly genome-wide coverage by generating knockout/knockdown mutant strain libraries for all non-essential genes in an organism (Oh *et al.*, 2010). These libraries are then further subjected to a large number of environmental conditions and stresses—with the observable in each of the settings being the fitness of individual clones (collected in stationary phase). Pairing the resulting data with an appropriate model of gene expression then allows for the inference of the underlying gene regulatory networks through estimation of significant interaction terms, along with those for production, degradation, expression level, etc. Although potentially applicable to any observable type, this approach may be particularly well-suited for the use with fitness data to help constrain any inferred gene regulatory network solutions to those dynamic modes that are most important for a given set of biological functions and conditions—e.g. growth on specific substrates or tolerance to certain stresses. Recent works have indeed suggested that the use of genome-wide fitness data can provide new perspectives on systems-level organization of cells and uncover novel gene regulatory interactions when compared with gene expression-based analysis (Hillenmeyer *et al.*, 2008, 2010); Deutschbauer *et al.*, 2011). Yet, although on a limited scale the idea of biological network characterization based on knockout data has been considered before, e.g. Winzeler *et al.* (1999), the emergence of high-throughput genome-wide gene deletion/interruption technology along with the use of population fitness rather than gene expression as an observable offers novel challenges as well as benefits to the task of gene network inference. On the one hand, microarray and other gene expression experiments typically generate high-dimensional data in the form of a real vector that comprises expression levels of multiple genes at each sampled time and/or condition point, whereas fitness measurements map the state of the system into a much lower dimensional space—e.g. that of a single real variable, such as growth rate. This inevitably leads to significant loss of information. On the other hand, deletion experiments usually involve simply cultivating and observing cells, which could be performed on a substantially larger scale, much more efficiently and under significantly greater range of conditions when compared with the relatively demanding gene expression assays. The ensuing ability to perform experiments simultaneously across the entire mutant collection substantially increases the overall genome-wide fitness data dimensionality—often putting it on par with available gene expression datasets. Furthermore, fitness observations allow for the preferential selection or overweighting of clones that display a desirable phenotype, e.g. the stronger the selection—the more significant the contribution of surviving strains. This becomes an increasingly important factor in many biotechnological and biomedical applications, whereby the contribution of practically irrelevant genes is effectively being filtered out—regardless of their statistical significance or dynamic state. Indeed, it has been shown that this type of data is very useful for the determination of target gene functions (Deutschbauer *et al.*, 2002, 2011; Hillenmeyer *et al.*, 2010; Pierce *et al.*, 2009; Steinmetz *et al.*, 2002).

Few systematic models and/or inference algorithms have been proposed for the elucidation of regulatory networks from fitness data. Conclusions are often being made on the basis of visual inspections or similar relatively naive strategies. Yet, greater prominence and availability of such data along with indications that fitness profiling might contain information about gene regulation suggest the need for a more comprehensive and rigorous inference approach. The analysis of ample data provided by genome-wide fitness experiments may also be useful in complementing network inference methods based on microarray gene expression and such other data by helping to initialize them or further refine their results.

In this work, we propose an algorithm for inferring gene regulatory networks from genome-wide knockout fitness data. Our approach is based on describing biological networks via a non-linear dynamical model and then elucidating model parameters from fitness measurements. The resulting parameter set can be used to identify the underlying regulatory network structure as well as to make forward-looking estimates of its function under temporal dynamics or environmental changes. In Section 2, we describe the system model and problem formulation. In Section 2.2, we provide a heuristic sample ordering selection algorithm based on correlation score to cope with the order selection problem that arises when using mutant fitness data. In Section 3, we describe the parameter estimation algorithm based on the unscented Kalman filter (UKF) technique. In Section 4, we use the proposed algorithm to analyse both a synthetic example as well as experimental data from yeast *Saccharomyces cerevisiae* and bacteria *Shewanella oneidensis*, which are further compared against known empirical results. We conclude the article with a summary and remarks regarding the proposed model and inference algorithm.

## 2 SYSTEM MODEL AND PROBLEM FORMULATION

### 2.1 System model

The outline of our approach is to describe a gene expression data model and to then extend it towards accommodating the observables supplied in the form of a large-scale knockout strain fitness dataset. To this end, we first introduce a basic model of gene expression as a weighted sum of (non-linear) functions of other genes with additive noise. We then obtain, through the removal of individual genes, the knockout strain network models used to drive the observed fitness model, which thus contains latent variables of our overall model, as described next in further detail.

Consider a gene regulatory network with total 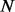 genes. Let 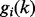, 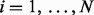, 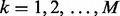 denote the gene expression level for the 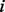-th gene at time 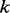. We denote observation or measurement data, 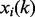, for 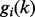 at time 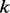 as: (1)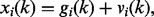 where 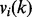 is the observation noise at time 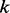 for 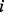-th gene. (Note that here the term ‘time’ is used in the generalized Bayesian inference sense and so may be loosely viewed as a discrete index enumerating individual experiments—rather than some continuous parameter of a kinetic biochemical system. Accordingly, the dynamic model we use is one based on ‘discrete time’, which thus fundamentally does not *a priori* assume or require continuity of states or observables.) We denote gene expression levels within the network by vector 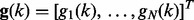, the observation vector by 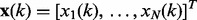 and noise vector by 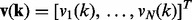. We assume that all vectors 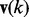 for 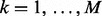 are independent and jointly Gaussian with zero mean and variance matrix 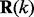. We approximate any multivariate gene–gene interactions by a combination of a linear expansion around the stationary solution and univariate non-linear terms. Specifically, we follow a discrete-time regulation model proposed in (Chen and Aihara, 1997) and describe the regulatory functions among genes as:
(2)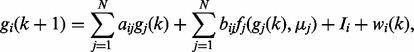 for 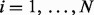, where 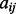 denotes the linear regulation coefficient from gene 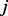 to gene 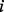 and 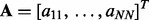; 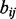 denotes the non-linear regulation coefficient from gene 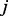 to gene 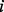 and 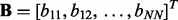; 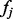 is the non-linear function for gene 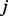 which is given by: (3)
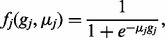
 where 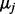 is the parameter to be inferred and 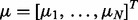; 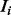 denotes the system expression bias for 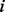-th gene and 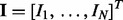, which will be inferred later. The noise vectors 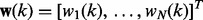 for 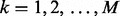 are assumed to be jointly Gaussian with zero mean and variance 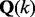. We also assume that they are independent from all 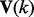. The regulatory network is realized as a state–space model, where we view gene expression levels as states and measurements as observations. The goal of inference is to estimate all the unknown parameters in the model. Inference results then provide estimates for all regulatory relations across the network.

Note that Equations (1) and (2) provide the description of the system in a manner most commensurate with expression data. We now proceed to extend this model to accommodate fitness data. Without loss of generality, we consider the case of a mutant library with single gene knockout per strain (with multiple-knockout collections being handled analogously, as discussed later) and assume that 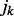-th gene has been deleted when the system is at time 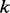. Note that ‘time’ here corresponds to the experiment number, with the index 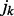 being determined as discussed in the previous paragraph. For the purposes of the single-knockout state–space model, expressions of all genes evolve without participation of gene 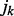. Therefore we set all 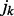 regulatory coefficients to zero. The states and system coefficients equations can then be summarized as: (4)
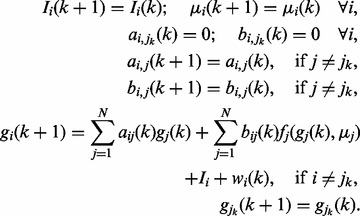



The last two equations determine the current system coefficients, which will be appended to the system state. Unlike the case of expression microarrays, here, each gene deletion/interruption strain measurement quantifies a single system property which is a function of all the remaining genes. In this article, we assume this measurement is a real number, which represents the fitness of the remaining network. (The model can be easily adapted into higher dimensional measurement case by direct extension.) Therefore, the observation 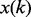 becomes: (5)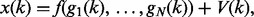 where 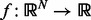 is typically not known *a priori*. We denote 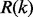 as the variance of 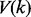 as before.

Various bases could be used to estimate 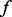 and associated coefficients. For instance, one could use a simple basis such as power series—i.e. a Taylor expansion. However, the speed of convergence for Taylor expansion is slow, resulting in a large number of parameters to be estimated. In contrast, the radial basis approach has been shown to be more robust and adaptive than Taylor expansion (Jiang *et al.*, 2003). Furthermore, it has been shown that with certain additional assumptions, the approximation by radial basis will converge to the true function in 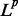 sense (Powell, 1987). Thus, we approximate 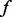 as: (6)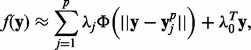 where 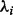 for 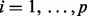 and 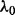 are the centres of the basis with 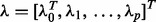; 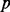 is the total number of basis functions used, which is a fixed constant; 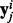, 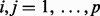 are the centre points of the waveform, which are chosen *a priori*; and 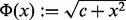
—the Hardy multi-quadratic function with constant 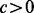, which serves as a classical choice for efficient radial basis expansion (Buhmann, 2003).

All the coefficients 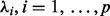 as well as 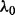 are parameters to be inferred and so are appended to the state variable. Therefore, the new augmented model state variable for knockout fitness data is: (7)
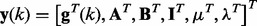



As a summary, the dynamical model we propose for regulatory networks with gene deletion/interruption mutant fitness data is: (8)
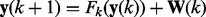


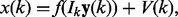
 where 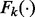 is the system function described in (4); 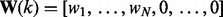 is the augmented noise vector; 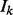 is the selection matrix, i.e. 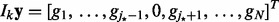, where the index 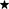 is determined by the time index 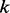; 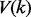 is the Gaussian noise as assumed before.

### 2.2 Data feeding order score

To infer the model given in (8), one needs to specify the order in which data are supplied to the algorithm. The question in what order data should be optimally fed into the inference algorithm thus arises. As this problem is fundamentally associated with the network structure itself, it can be solved exactly only if the structure of the network is already known. (And even then, the problem typically has NP-hard complexity since one needs to test all possible permutations.) Since the feeding order will have direct impact on the performance of the inference result, we still need a strategy to find an ‘optimal’ feeding order without explicitly exploring all potential network structures. In this section, we propose a heuristic strategy based on correlation score, which is then compared against selecting the feeding order randomly and shown to be more ‘optimal’ by offering certain advantages.

The basic idea behind the proposed heuristic is that we should feed the most useful data first. In this work, we use correlation as a measure of such ‘usefulness’. The intuition is that for sequential inference of the model (8), the data should be fed based on their importance in a certain sense. The reasoning for this strategy is that, in sequential inference, a good starting point usually provides a superior opportunity to converge to a good result and vice versa. Moreover, once we have already fallen into a steady state or an attracting basin, subsequent data may have less influence on the final result, since it may be difficult to jump away from the local attractor. By contrast, at early stages, this influence may be vital to the final inference result. Heuristically, the importance of a certain sample may be determined by the connectivity of the deleted gene. If it has many connections to other genes, it may likely play an important role in the network, which would be reflected in the measurement value (e.g. fitness). Based on this approach, the feeding order for a sample is related to the importance or connectivity of the corresponding deleted gene, which could be quantified by using correlation as a metric.

Specifically, we consider fitness observations 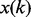, 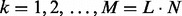, where 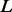 is a positive integer representing the total rounds of experiments. Note that without loss of generality, we can always assume that 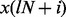, 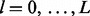, 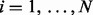 is the 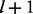 round fitness data for gene 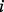. The score 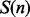 for gene 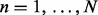 is calculated as: (9)
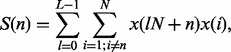
 with gene feeding arranged in the order of descending 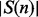. The summation over 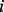 calculates the correlation under round 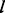. The final score is the summation over all rounds. This approach may be compared with choosing the feeding order randomly, as will be done later using concrete examples.

Finally, note that a similar feeding heuristic may be used to optimize the order of individual experimental conditions as well. The utility of this additional step, however, needs to be weight against the diminished significance of individual permutations among observation data points as the number of conditions becomes large as well as the increasing computational overhead this may entail. As in our applications the number of conditions was substantially greater than either the number of genes or the number of connections between them, we found the additional computational costs such extra step would entail to be unwarranted.

## 3 THE UNSCENTED KALMAN FILTER APPROACH FOR INFERENCE

In the previous section, we proposed a system model for describing gene regulatory networks at the state–space level. The approach now requires an algorithm for estimating the unknown parameters of the model, from which network organization and other biological system properties may be inferred. Here, a Kalman filter technique—a well-characterized estimation strategy for elucidating state-space models of regulatory networks (Wang *et al.*, 2006, 2009), and specifically, an unscented Kalman filter (UKF)—is used to accomplish this task. UKF is used to estimate all the parameters in order to cope with the expected non-linearity of the model (Julier and Uhlmann, 1997), as it has been shown to have superior performance when compared with traditional approaches, such as the extended Kalman filter, especially with availability of enough data.

Classical Kalman filter technique iteratively uses innovations in state and measurement predictions—updating the system sequentially (Simon, 2006). The general idea of a Kalman filter can be summarized as:
(10)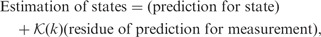 where 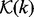 is the ‘gain’ for the residue. The original approach by Kalman is based on linear differential equation model under Gaussian noise assumption. An extended Kalman filter (EKF) has been proposed for dealing with non-linear models (Corigliano and Mariani, 2004). The idea of EKF is to linearize the non-linear function by approximating it with the first-order Taylor expansion. However, such an approximation is quite coarse and insufficient under general circumstances. Some approaches look to remedying the situation by using higher-order terms Taylor expansion terms, which—while more accurate—generally leads to dramatic increases in complexity (Daum, 2005). Alternatively, UKF approximates the non-linear function by viewing it as a non-linear transform and then using the so-called ‘sigma points’ to capture the posterior mean and covariance accurately up to the third order. Compared with EKF, UKF provides a more accurate approximation without significant increase in complexity and has been shown superior in many practical situations (Wan and Van Der Merwe, 2000). Another advantage of UKF is that it does not require the calculation of model’s Jacobian or Hessian, which makes the algorithm and associated mathematical derivations less involved (see more below).

As noted, the UKF is based on the idea of choosing sigma points from the unscented transform. Consider a random vector 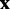 being passed through a non-linear transform 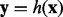. In order to calculate the mean and variance of 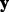, we choose the *s*igma points 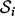, 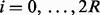 and their weights 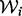 as follows: 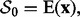 {\cal S}_0
= {\rm E}({\bf x}), 
(11)
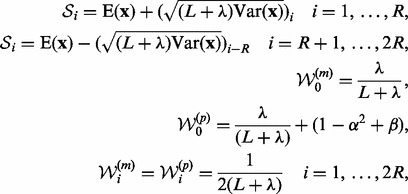
 where 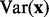 is the variance matrix of the random variable 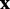; 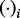 denotes the 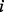-th column of the input matrix; 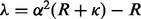 is the scaling parameter; and 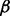 is a parameter incorporating prior knowledge of 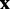. Under Gaussian noise assumption, we can set 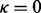, 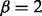, and 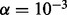 (Julier and Uhlmann, 1997).

After computing all the sigma points 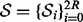 and their corresponding weights, the mean and variance of 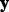 can be approximated as: (12)
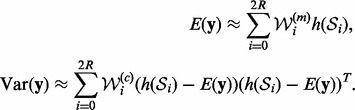



In order to infer the dynamical system model described in (8), we simply concatenate the state variable 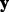 with the noise vectors 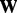 and 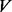 to form a new augmented vector (13)
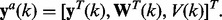



Viewing the 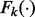 and 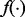 in (8) as non-linear transforms allows us to calculate the corresponding sigma points as well as to approximate their mean and variance for use in sequential updates.

We summarize the UKF-based algorithm for inferring model (8) from knockout fitness data in Section S1 of the Supplementary Material.

## 4 RESULTS

### 4.1 Inference of synthetic network

**Fig. 1.**
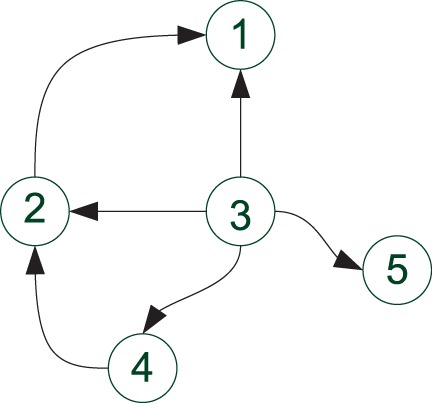
Structure of the synthetic network

In this section, we investigate the performance of the proposed algorithm for inference of a synthetic network. The network has both linear and non-linear connections with the graph structure specified in Figure 1. The dynamics of the network are based on the proposed model (8), with arrows denoting the direction of regulatory interactions. The parameters of the network are given in Table 1, with the variance of the model noise 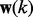 taken as 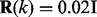 for 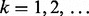, where 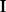 is the identity matrix. We also take the variance of measurement noise (error) 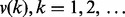 in (1) to be 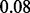. The deletion data are obtained by sequentially removing each gene and its corresponding connections. We use 10 rounds of sample data, i.e. 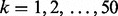. Finally, five basis functions are included in the fitness model. (Although there is always a trade-off between accuracy and complexity for number of basis functions to be used the dimension of the problem is proportional to the number of basis functions. The number of basis functions may be chosen quantitatively, if needed—e.g. by cross-validation—but here five basis functions appears to be sufficient.) The fitness function model 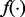 in (5), thus becomes: (14)
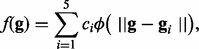

where 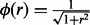 with 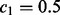, 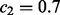, 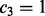, 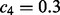, 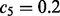; and 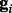 being 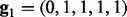, 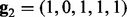, 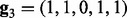, 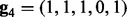 and 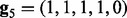.

**Table 1.** Comparison of linear coefficients (LCs) and non-linear coefficients (NLCs) of the inferred regulatory network and the underlying model system

Edge	Synthetic LC	Synthetic NLC	Inferred LC	Inferred NLC
(2,1)	0.7	0.5	0.621	0.781
(3,1)	0.7	0.5	0.491	—
(3,2)	0.7	0.5	1.091	—
(3,4)	0.7	0.5	0.861	1.132
(3,5)	0.7	0.5	0.682	0.581
(4,2)	0.7	0.5	—	—
(5,4)	—	—	0.981	0.852

Following the proposed algorithm, we first determine the ‘optimal’ data feeding order using the correlation score described in Section 2.2. The resulting order is 3, 2, 1, 4, 5. We can see that this ordering generally coincides with the importance of each node in the sense of node connectivity. This further reaffirms the validity of the proposed heuristic that the data from most connected nodes should be fed in first.

**Fig. 2.**
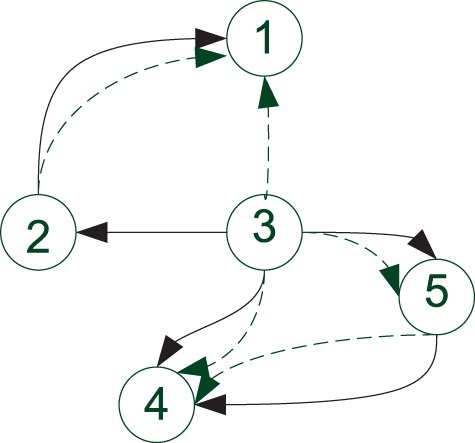
Connection structure of the inferred synthetic network. Linear connections are denoted by solid lines. Non-linear connections are denoted by dashed lines

Following subsequent steps, we infer parameters of the underlying network, which are then filtered to remove values below noise threshold set at 40% of their maximal variation (0.431 and 0.443 for linear and non-linear coefficients, respectively). The resulting inferred synthetic network is shown in Figure 2, with regulatory interaction parameters given in Table 1.

Comparing the inferred network to the true model, we can see that the elucidated results correctly identify the presence of five out of six regulatory interactions and suggest one non-existent one (false negative rate of 16.7% and false positive rate of 8%, respectively, accounting for the direction of regulation). If we account for both linear and non-linear connections individually, a further two connections are not discovered (linear 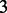 to 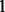 and non-linear 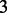 to 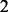).

**Fig. 3.**
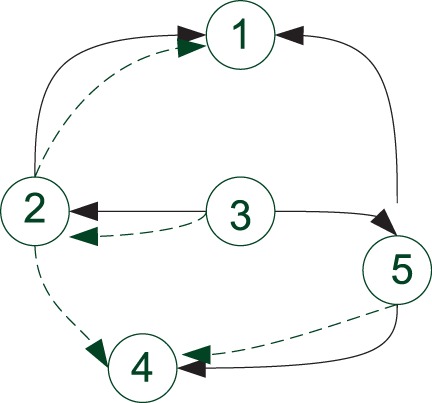
Connection structure of the inferred network without using the optimal feeding order. Here, the original data order 1, 2, 3, 4, 5 is used instead of the optimal one 3, 2, 1, 4, 5 (which is used in previous figures). Linear connections are denoted by solid lines. Non-linear connections are denoted by dashed lines

Finally, we have compared the effect of choosing the feeding order according to the prescription provided in Section 2.2 versus selecting it randomly. As can be seen in Figure 3, using the original order 1, 2, 3, 4, 5 instead of the optimal one 3, 2, 1, 4, 5 results in a significant degradation of inference results.

To quantify this effect more rigorously, we define missing rate 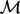 as the difference between 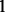 and the ratio of the number of correctly identified edges to the total number of edges in the synthetic network, i.e. 12 here. We also define false rate 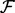 as the ratio of number of incorrectly identified edges to the total number of edges not in the synthetic network, i.e. 228 here. Then, for the inference result using the optimal order, we have 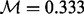 and 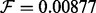. In contrast, using the original order yields, 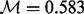 and 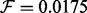.

Table 2 summarizes the results when using the optimal order versus the average of 30 randomly chosen orders. As we can see, the optimal order has performed better than randomly chosen orders in both the missing and false rates.

**Table 2.** Inference algorithm performance when using the optimal feeding order versus the average of 30 randomly chosen orders (lower is better)

Order	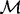	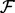
Optimal order	0.333	0.00877
Average of random chosen orders	0.753	0.0729

### 4.2 Inference of *S.cerevisiae* GAL network

**Fig. 4.**
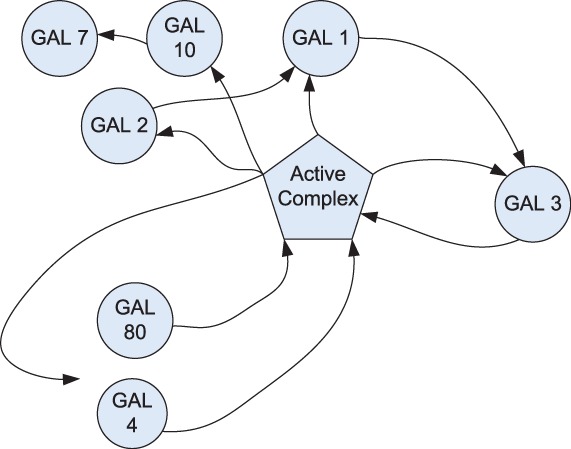
Structure of the empirical *GAL* network

We now apply the described inference algorithm to *GAL* regulatory network that controls galactose utilization in yeast *Saccharomyces cerevisiae*. *GAL* regulation represents one of the most historically prominent model systems in yeast because of its importance for the studies of eukaryotic regulation and relatively self-contained nature. Figure 4 summarizes the empirical knowledge of *GAL* network structure (Egriboz *et al.*, 2011; Flick and Johnston, 1990; Johnston *et al.*, 1994; Lohr *et al.*, 1995; Ostergaard *et al.*, 2000).

**Fig. 5.**
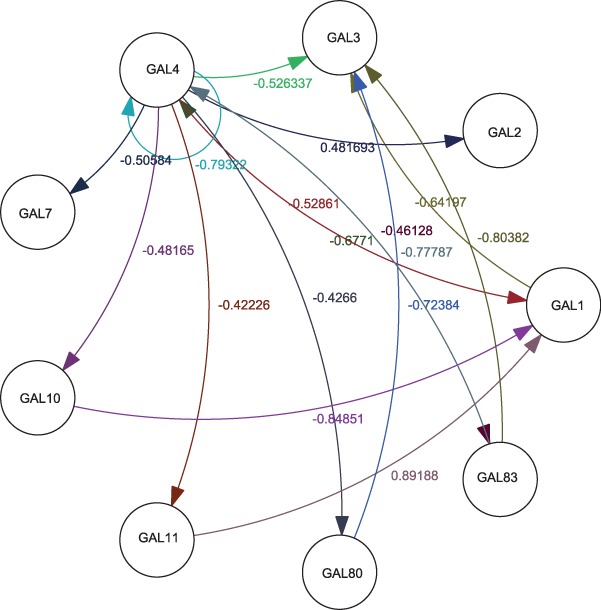
Linear structure of the inferred *GAL* network

**Fig. 6.**
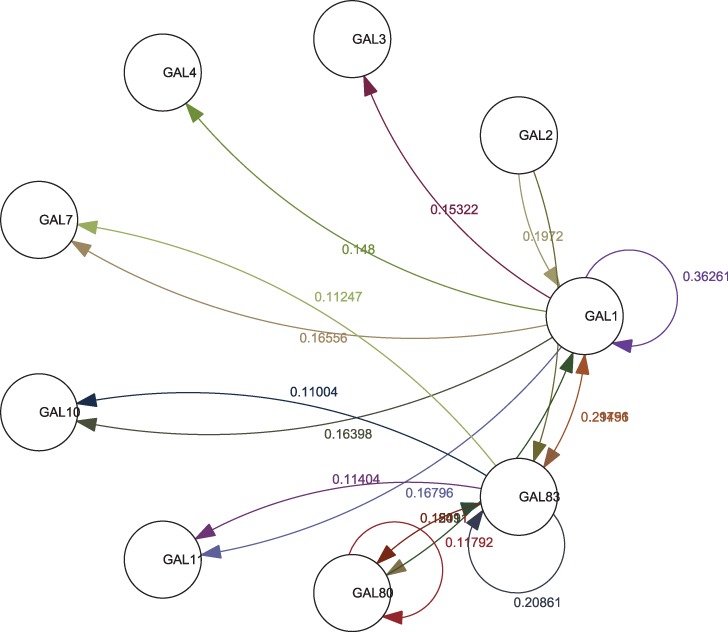
Non-linear structure of the inferred *GAL* network

**Fig. 7.**
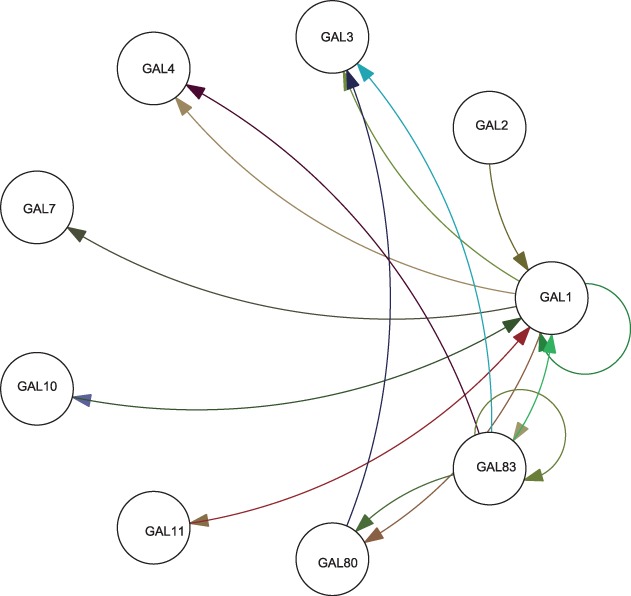
Combined structure of the inferred *GAL* network

Our analysis is based on yeast deletion strains fitness data previously collected by Giaever and co-workers (Giaever *et al.*, 2002). The one-dimensional measurements were performed under various environmental conditions—such as different concentrations of galactose, alkali, sodium chloride, sorbitol, etc. We utilize nine sets of samples from different environmental conditions for network inference using the described algorithm, with 40% variation threshold as before. The nine sets of samples are formed by combining arbitrary samples from each environmental condition as representatives. Figures 5 and 6 show the inferred network graph structures as identified by linear and non-linear coefficients, respectively. A combined linear–non-linear network graph is given in Figure 7 (note, in the interest of clarity, edge weight labels and lower-weight edges have been removed).

It could be seen from Figures 5 and 6 that *GAL 1*, *3*, *4* and *80* have the most connections and the largest coefficients, which is in accord with the known fact that these are the regulatory genes in the network, with others being regarded as the structural genes. Additionally, we note that *GAL 80* has negative connections to *GAL 3* and *GAL 4* as well as that *GAL 4* has negative connections to *GAL 1* and *7*, all of which coincides with the empirically known fact that *GAL 80* negatively regulates *GAL 3*, *4* and that *GAL 4* leads to the repression of transcription from *GAL 1*, *7*. The connections between *GAL 1*, *2* and *GAL 3* also reflect the fact that *GAL 2* and *GAL 1* regulate *GAL 3* by protein utilization pathway. Finally, we see that there is no direct connection from *GAL 11* to *GAL 80,* which also coincides with the fact that *GAL 11* does not have direct interaction with *GAL 80*. On the other hand, we find that although inference results discover the connections between *GAL 3* and *GAL 80* as well as *GAL 3* and *GAL 4,* they may not be in the correct orientation. Otherwise, inferred influences among regulatory genes appear to be in a general agreement with empirical understanding of the system.

### 4.3 Inference of *Shewanella TyrR–LiuR* network

**Fig. 8.**
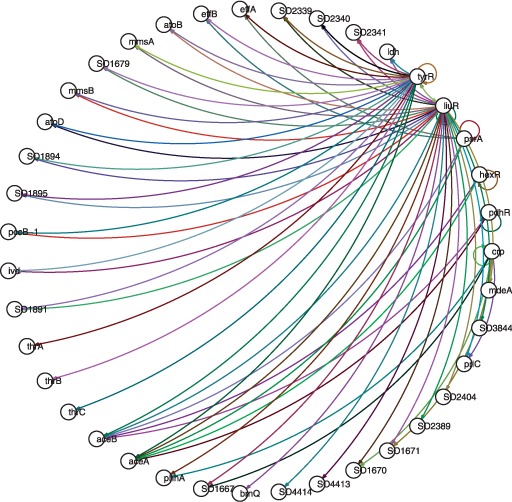
Structure of the empirical *TyrR–LiuR* network

**Fig. 9.**
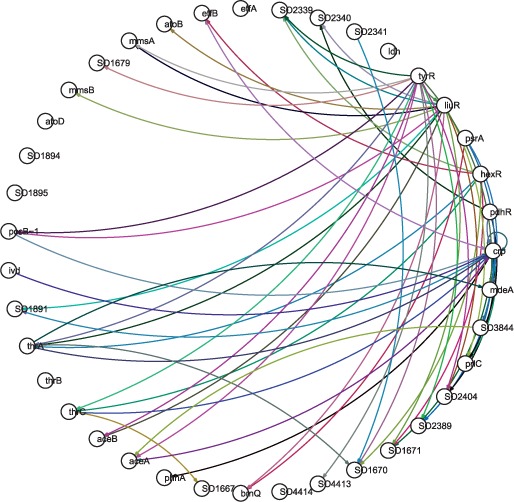
Linear structure of the inferred *TyrR–LiuR* network

**Fig. 10.**
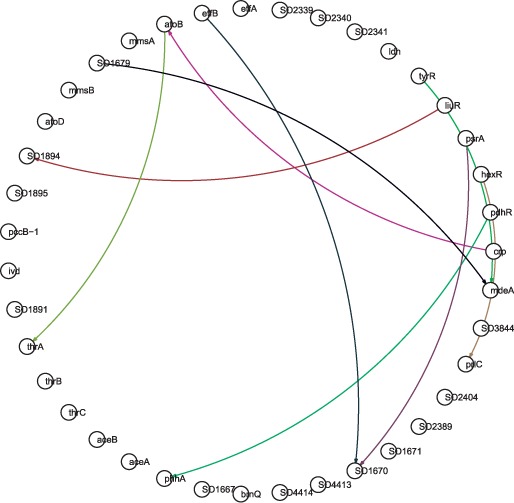
Non-linear structure of the inferred *TyrR–LiuR* network

In this section, we apply our algorithm to the *TyrR–LiuR* amino acid utilization–degradation network of *S.oneidensis* strain MR-1. Its ‘true’ structure, shown in Figure 8, was derived from hand-curated high-confidence regulatory interactions catalogued at MicrobesOnline and RegPrecise (Dehal *et al.*, 2009; Novichkov *et al.*, 2010). Inference was performed by using the proposed algorithm on 287 sets of fitness data (number of fitness measurements for each of the knockout strains under different growth conditions), with results shown in Figures 9 and 10. The inference results are compared to and seen to be in general correspondence with the true structure, Table 3.

**Table 3.** Comparison of Ground truth to the Inference algorithm performance

Number of edges (Ground truth)	Number of edges (Inference)	Number of correctly identified edges
57	69	25

## 5 DISCUSSION

We have proposed a dynamical model and an algorithm for inference of gene regulatory networks based on genome-wide knockout fitness data—an emerging data type, whose utility in biological systems identification has not been sufficiently explored to date. The algorithm uses a state–space model to capture the dynamical and non-linear nature of such networks. An unscented Kalman filter is used to infer the unknown parameters in order to cope with model non-linearity.

Although fitness data inherently suffers from loss of information caused by its reduced dimensionality, when compared with the more widely explored gene expression data type, the potentially larger amounts of and more contextually/phenotypically meaningful data provided may be able to compensate for the relative lack of resolution as our work appears to suggest. The analysis of a synthetic example as well as empirical *GAL* and *TyrR–LiuR* network data presented here shows that the described algorithm is able to provide satisfying inference results even for relatively complex mechanisms.

Ultimately, the two data types—gene expression and knockout fitness—may be expected to be most informative when used in a complementary fashion. As noted previously, inferences generated from genome-wide knockout fitness data could be used to facilitate network elucidation methods based on gene expression by helping initialize or further refine their predictions. Conversely, information provided by gene expression data may be exploited by the proposed algorithm in conjunction with knockout fitness data to synergistically improve final inference results. For instance, gene expression data may be collected under a more limited set of conditions, for a subset of the mutant library, or just for the wild-type strain; and used for the initial inference of an augmented state vector 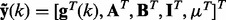
—associated only with the gene expression part of the model, Equations (1)–(4). Such preliminary results could then be used as a prior for the subsequent network inference round, which uses genome-wide fitness data in order to take advantage of its potentially larger scale or more immediate availability across a range of conditions, as well as to introduce corresponding phenotypically significant refinements (as discussed earlier).

Several other directions appear promising toward potentially further extending and improving the inference methodology proposed here. For instance, the non-linear univariate interaction model, Equation (2), may be augmented with explicitly multivariate terms. This should serve to enhance resolution of non-linear interactions for a given gene across multiple reaction partners and improve modelling accuracy, though at a cost of substantial computational overhead owing to complications related to multiplicative noise propagation and the need to account for intermediate molecular complexes. Perhaps a more straightforward approach involves using the model to accommodate multiple-knockout experiments (i.e. those involving multiple inactivated genes in each strain). This is done analogously to the way single-knockout networks have been analyzed here by simply setting all of the corresponding interaction term coefficients to zero for the genes in question. The reason we have focused less on such applications in this work, however, is the present scarcity of multiple- versus single-knockout observation data. Finally, additional constraints could be incorporated to help account for other pre-existing sources of experimental or heuristic information. For example, we have noted earlier the possibility of applying feed optimization schemes to condition data ordering. One may further look at various sparseness conditions as a way of improving computational efficiency and incorporating pre-existing knowledge about the network during inference. [Though, care should be taken, as significant variations in effective regulatory network topology may naturally arise across experimental conditions or in going from one related organism to another (Bergmann *et al.*, 2003; Luscombe *et al.*, 2004)]

Overall, we believe that these questions help further support the suggestion that the analysis of genome-wide fitness data (whether directly or in conjunction with gene expression data) towards understanding of biological systems function and, in particular, inference of gene regulatory network organization offers a rich new area of future research—to which this article is seeking to make an initial contribution.
